# *Invivo* and *in vitro* analyses of surface whitening in hydrophobic trifocal intraocular lenses

**DOI:** 10.3389/fmed.2025.1518707

**Published:** 2025-03-18

**Authors:** Margarita Cabanás, Jorge Navalón, Rafael Luchena, William Lee, Alejandro Cerviño

**Affiliations:** ^1^Clínica Baviera Sevilla, Sevilla, Spain; ^2^Department of Ophthalmology, Hospital Virgen del Rocío, Sevilla, Spain; ^3^Clínica Baviera Valencia, Valencia, Spain; ^4^AST Products, Inc., Billerica, MA, United States; ^5^Department of Optics & Optometry & Vision Sciences, University of Valencia, Valencia, Spain

**Keywords:** multifocal IOLs, hydrophobic IOL, optical quality, IOL transparency, subsurface nanoglistening

## Abstract

**Introduction:**

Transparency loss due to the whitening of trifocal hydrophobic intraocular lenses (IOLs) is a rare but poorly understood phenomenon. This study investigates its characteristics, underlying mechanisms, and clinical impact to determine its relevance for patient care.

**Methods:**

A clinical and laboratory analysis was conducted on affected IOLs. Two patients with bilateral trifocal IOL implantation, where one eye exhibited whitening, underwent visual performance testing, light distortion assessment, optical coherence tomography, Scheimpflug imaging, and patient-reported outcome evaluations. An explanted IOL was examined using microscopic and optical bench methods, and attempts were made to replicate the whitening process *in vitro* using unused control lenses.

**Results:**

Clinical findings showed that IOL whitening had minimal impact on visual acuity and light distortion, with no significant impairment reported in patient-reported outcomes. Imaging and laboratory analysis suggested that whitening is caused by subsurface nanoglistening (SSNG), a previously unreported phenomenon in this IOL material. The modulation transfer function of the affected IOLs remained comparable to control lenses, and *in vitro* attempts to induce whitening were unsuccessful.

**Discussion:**

This study provides the first evidence of SSNG in this hydrophobic IOL material, demonstrating that while whitening can occur, its clinical significance is generally low. Given the predominantly subjective nature of symptoms, management should be individualized, with explantation decisions made in close consultation with the patient. These findings contribute to a better understanding of IOL material stability and patient counseling in cases of transparency loss.

## 1 Introduction

Intraocular lens (IOL) opacifications represent a significant concern in the realm of ophthalmology, and despite advancements in surgical techniques and materials, the development of opacifications remains a persistent issue.

IOLs are mainly made from hydrophobic or hydrophilic acrylate, PMMA, and silicone. Opacifications in IOLs include posterior capsule opacification (PCO), biological deposits, calcification, decoloration, or material transparency loss. Transparency loss can be, in turn, due to glistening, caused by fluid-filled microvacuoles in the optical zone that glisten in light, and subsurface nanoglistening (SSNG), resulting in reflected white light due to light scattering from nanosized fluid-filled microvacuoles on the IOL surfaces (1). Different materials have specific opacification patterns, with hydrophobic acrylic lenses prone to glistening-like transparency loss, while calcifications are more common in hydrophilic acrylic lenses (2).

Hydrophobic acrylic lenses are popular due to their ease of insertion, good vision restoration, and reduced PCO incidence (3, 4). However, loss of transparency events has been reported in some studies (1, 5, 6). Glistening or SSNG can gradually develop, potentially affecting visual quality over time due to increased intraocular light scatter (7). While this theoretical impact in visual symptoms and performance has been reported by some authors (8), there is controversy as other studies did not find significant clinical effects even in the long term (9, 10).

The present study analyzed *in vivo* and *in vitro* diffuse IOL whitening detected in routine postsurgery assessment a few months after implantation of a new glistening-free material, trifocal design IOLs. The analysis aims to describe the nature of the transparency loss, establish a hypothesis of the likely cause, and determine its clinical impact.

## 2 Methods

This study was approved by the Institutional Review Board of Clinica Baviera, Spain, and written informed consent for publication was obtained from the affected patients.

Three patients who received bilateral implantation of the same hydrophobic, low water content, biaspheric design trifocal intraocular lens were included in the study (all female, aged between 50 and 73 years). Only one eye in each patient showed IOL whitening, while the fellow eye remained unaffected. These are the only known instances of this specific IOL material with spontaneous loss of transparency to date. Whitening occurred between 2 weeks and 3 months post-implantation, with surgeries being uneventful. Routine postoperative follow-up showed no significant issues, except for one instance due to an episode of anterior uveitis approximately 2 months after implantation. The condition resolved satisfactorily with treatment. In this case, the affected IOLs were explanted whole and sent for laboratory testing, which occurred approximately 6 months postop.

The other two patients were examined *in vivo*, with data from the unaffected eye used as a control.

## 3 Intraocular LENS

All patients included in the study were bilaterally implanted with the Asqelio^™^ Trifocal TFLIO130C IOL (AST Products Inc., Billerica, MA, United States). This IOL has a biaspheric geometry with a posterior diffractive optic design (15 rings within the central 4.5 mm) in its 6.0 mm in diameter optical zone. It has a total diameter of 13.0 mm and provides an addition for near of +3.30 D and + 2.20 D for intermediate distance. The lens is built in powers ranging from +5.00 to +34.00D in 0.50 D increments, a C-Loop platform, and a light distribution among its foci of 50% for distance, 24% for intermediate, and 26% for near. It is made via lathe-cutting of soft hydrophobic acrylic material (glistening-free) with a refractive index of 1.50, water content below 0.5%, Abbe number of 50, and spherical aberration of −0.27 microns.

## 4 *In vitro* analysis

The *in vitro* laboratory analysis consisted of two different phases carried out at the R&D department of AST Products, Inc. (Billerica, MA, United States).

The first phase was the analysis of the explanted IOLs, in which the following procedures were carried out:

1. Microscopic analysis of the explanted IOL appearance and comparison with unused control IOLs. Microscopic analysis was carried out using a digital microscope consisting of a CCD camera (BV-2 K), a lens (COMPUTAR ALN-M2528-MPW3), and a monitor screen to image IOLs at approximately 20x magnification.
2. Optical bench analysis of the optical quality of the explanted IOLs and comparison with unused control IOLs were performed using PMTF equipment (Lambda-X, Nivelles, Belgium) to evaluate the modulation transfer function (MTF) and record USAAF charts for sensitivity comparison.

The second phase included opacification testing to investigate the potential cause of the loss of transparency when stressing unused control lenses. The opacification test is performed by (1) placing the unused IOLs in a saline container and the entire container is placed in a water bath at 42°C for 1 h; (2) thereafter, the saline and IOL container is placed in a water bath at 32°C for another hour (one cycle: 42°C → 32°C); (3) the unused IOLs are removed from the saline container, and a microscope image is captured; and (4) the procedure is repeated for five cycles.

A total of 13 unused control trifocal IOLs and 13 unused control monofocal IOLs were used for this purpose.

## 5 *In vivo* analysis

The clinical protocol consisted of one single visit approximately 9 months after implantation, in which the following procedures were carried out in both the affected and fellow eyes, which acted as control:

*Defocus curves* were obtained monocularly and binocularly under photopic conditions. The step size in diopters was 0.50 D, ranging from +1.00 to −4.00 D.

*Contrast sensitivity* was determined using the CSV1000 (VectorVision, Greenville, OH, United States) and the Cc100 (Topcon Europe Medical BV, The Netherlands). Contrast thresholds were determined monocularly at 3, 6, 12, and 18 cycles per degree (cpd) spatial frequencies. The results were plotted and compared between the affected and fellow eye on each case, as well as against reference values.

*Anterior segment optical coherence tomography (OCT)*, using the SOCT Copernicus REVO (Optopol Technology Sp., Zawiercie, Poland). The system is a spectral domain OCT that uses an 840 nm wavelength aimed at posterior segment imaging, which includes a built-in anterior segment lens that allows for collecting high-resolution anterior segment images. It features an anterior chamber exam that allows optimizing the view of the anterior chamber angle and IOLs, obtaining axial resolutions of 5 microns in tissue and lateral resolutions of 12–18 microns.

*Scheimpflug tomography imaging* was carried out using the Pentacam rotating Scheimpflug camera (Oculus Inc., Germany). This instrument uses the Scheimpflug principle to acquire cross-sectional images of the cornea and the lens. It also features a Scheimpflug-based optical densitometry analysis, which is able to objectively measure haziness by the assessment of the backscattered light.

*Light disturbance analysis* was assessed monocularly using the light distortion analyzer (LDA, CEORLab, ceorlab.wixsute.com). Light disturbance refers to a phenomenon characterized by the appearance of a halo around a central luminous point and serves as an indicator of visual quality. A detailed description of the system and measuring procedure can be found in the literature (11). In the present investigation, semi-meridians with an angular separation of 30 degrees were measured using an in-out routine.

*Patient-reported outcomes (PROs)* were determined through the CATQuest9SF questionnaire and a visual symptoms questionnaire.

## 6 Results

### 6.1 *In vitro* analysis

The IOLs were explanted on 4 January 2023, collected, and forwarded to the manufacturer for further investigation. Production and inspection records were reviewed, and no abnormalities were found. All the IOLs belonged to different batches.

### 6.2 Microscopy analysis

The IOLs were received in the laboratory in a semidry state, and no loss of transparency of any kind could be observed. After 1 h immersed in saline solution at 35°C, a partial diffuse loss of transparency became evident, identical to that observed *in vivo* on the day the IOLs were explanted.

The appearance is compatible with SSNG, also known as “whitening,” observed as white light reflection due to retroscatter of light when encounters fluid-filled microvacuoles just below the anterior and/or posterior IOL surfaces (1). It has also been described that the whitening disappears when the IOLs are in a dehydrated state, as occurred in the present case (12, 13).

**Figure 1:**
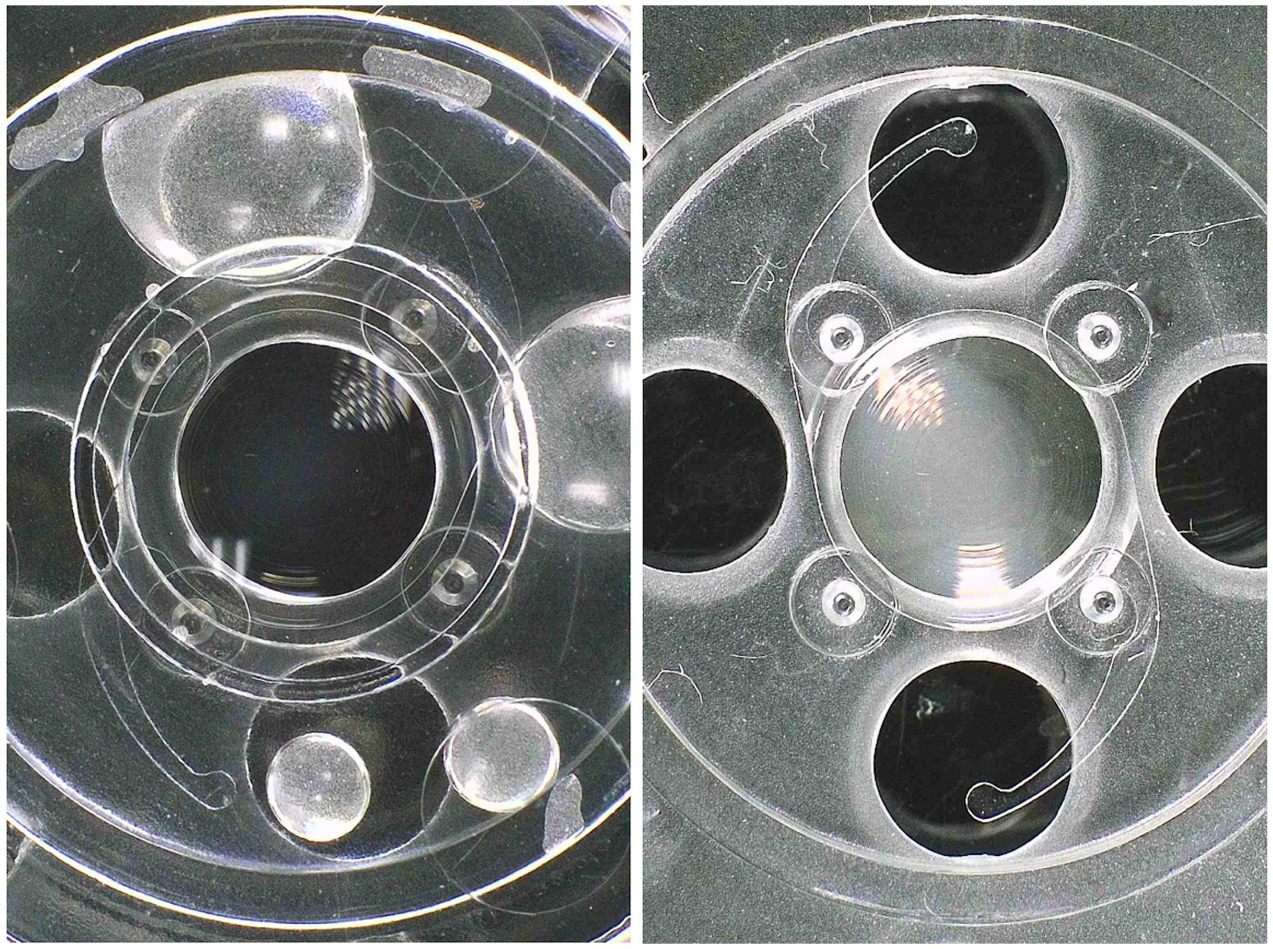
View through the microscope of the appearance of the explanted IOLs (right) compared to the unused control IOLs (left).

Microscopic analysis also excluded biological deposits on any of the IOL surfaces as potential causes of the loss of transparency. In Figure 1, the explanted IOLs observed through the microscope can be observed (right) compared to the unused control IOLs (left).

### 6.3 Optical bench MTF analysis

MTF values obtained from the explanted IOLs with whitening measured in the optical bench are similar to those obtained from an unused control IOLs (Table 1).

**Table 1:** MTF values obtained at the different vergences for the unused control trifocal IOL, the explanted IOLs prior to rehydration when the loss of transparency could not be seen (explanted IOL-D), and the explanted IOL rehydrated when loss of transparency was evident (explanted IOL-H).

MTF @50lp/mm	Far	Mid	Near	Total
Unused IOL	0.39	0.15	0.18	0.75
Explanted IOL-D	0.42	0.16	0.18	0.76
Explanted IOL-H	0.43	0.16	0.17	0.76

This has been reported in the literature, where optical simulations using ray tracing showed that MTF was not significantly affected by SSNG (14). Other optical bench studies also showed similar light transmission values, but greater dispersion in IOLs with SSNG compared to controls, although they indicated that retinal straylight values would not induce noticeable visual alterations (15). This will be assessed later in the *in vivo* analysis of light distortion and visual performance.

**Figure 2:**
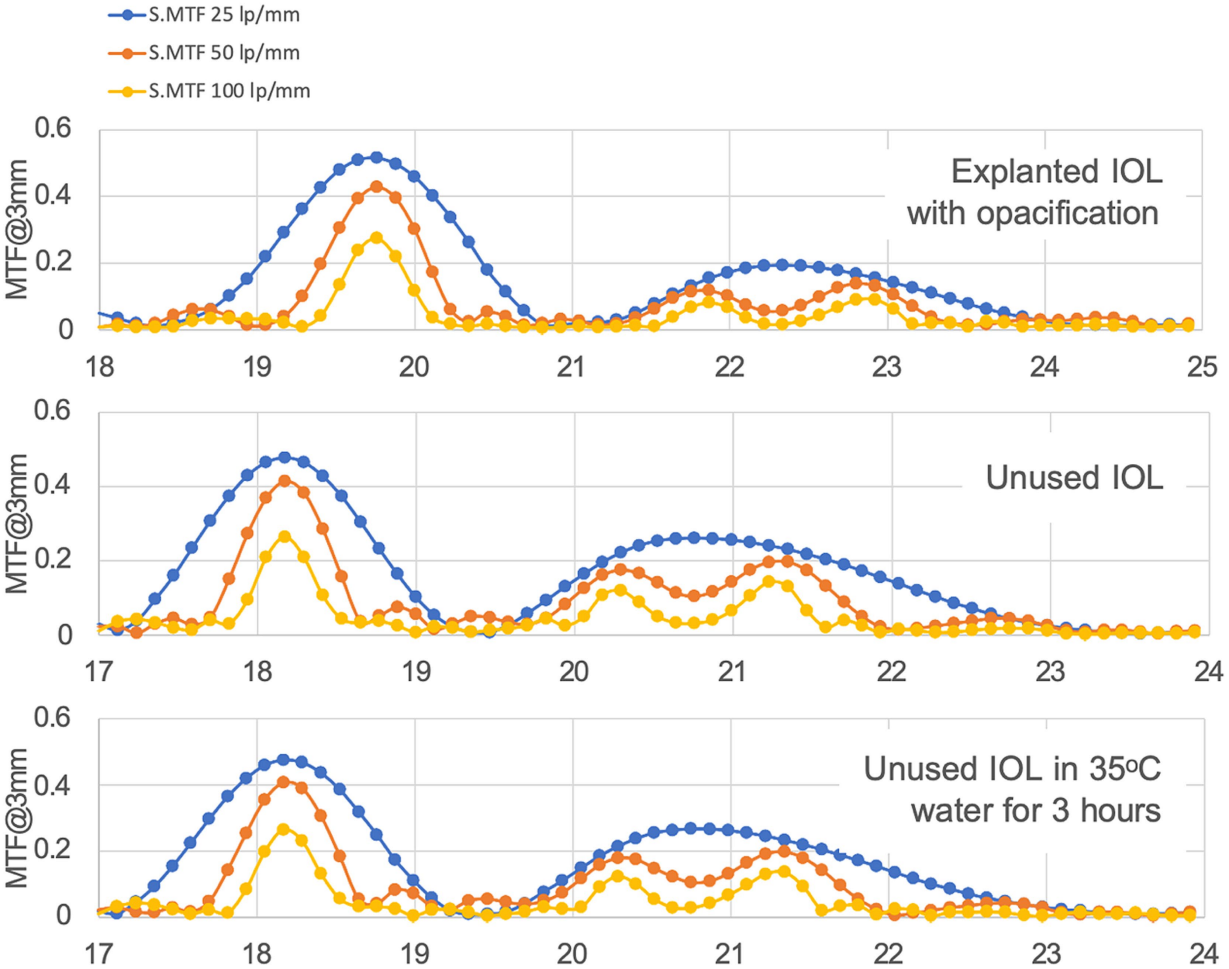
Through focus MTF at different resolutions for a 3 mm pupil. Horizontal axis displays optical power in diopters (D).

In addition, through-focus MTF profiles do not show a significant change between the affected IOL and control lens, with minor changes at intermediate and near foci (Figure 2).

**Figure 3:**
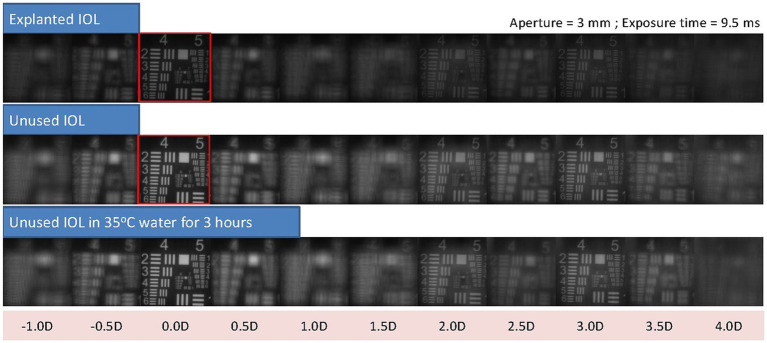
USAAF test simulation for different vergences from optical bench data shows the loss in contrast between the explanted IOLs and the control lens. As a reference, red squares highlight the USSAF target for distance through the explanted IOL and the control lens, where the difference in contrast, can be easily observed.

On the other hand, contrast is affected by whitening in this IOL by a 60% reduction, as may be observed in the United States of America Air Force (USAAF) test targets shown in Figure 3.

### 6.4 Opacification test

**Figure 4:**
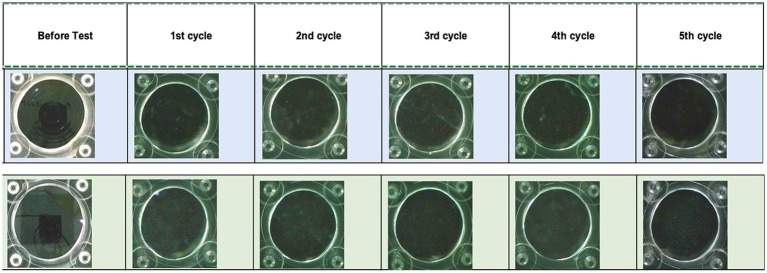
Microscopic images of two of the control IOLs, trifocal TFLIO130C (upper row) and monofocal (lower row), used for the opacification test before and after each of the cycles.

Figure 4 shows a sample of the captures taken of a trifocal and a monofocal after each of the five cycles. The polymer structure was not altered in any IOL, and no loss of transparency was generated.

### 6.5 *In vivo* analysis

**Figure 5:**
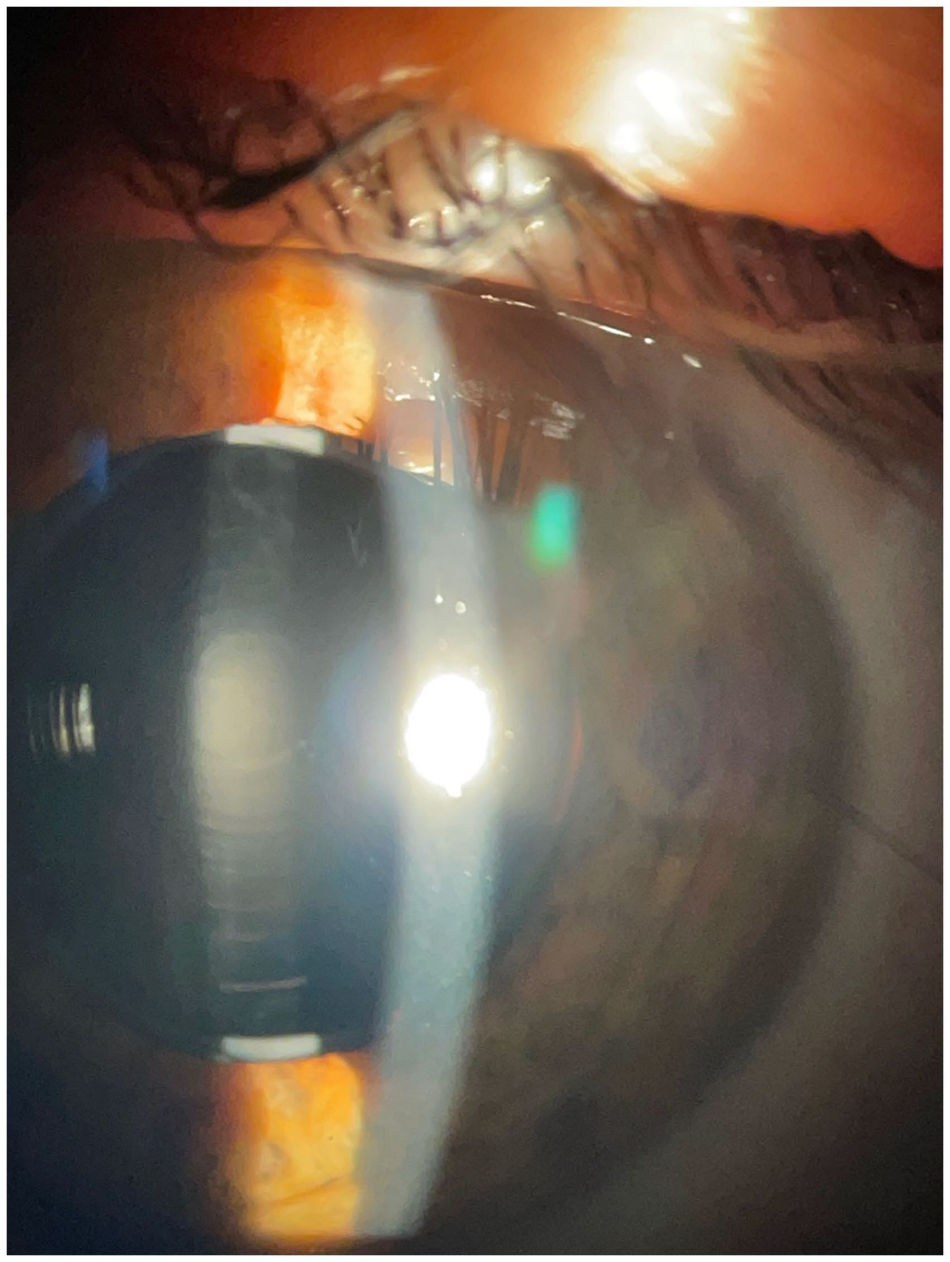
Diffuse whitening of the IOLs observed by direct illumination with the slit lamp.

Clinical observation of the IOL *in situ* with the slit lamp shows that IOL whitening is not visible with retroillumination techniques but becomes evident with direct illumination techniques such as Voigt parallelepiped with an illumination system forming a 30- to 60-degree angle (Figure 5). Kanclerz et al. (16) described the microscopic appearance of IOL opacifications and indicated that SSNGs may give the IOL surface a whitish coloration when the light is directed at the IOLs at an angle of incidence of 30 degrees or greater.

The whitening did not have any implications for ophthalmoscopy assessment with a Volk lens.

### 6.6 Defocus curve

Binocular defocus curves show good performance at all distances across the range from distance to near, with visual performance above 0.2 LogMAR across the whole range. Compared against reported binocular defocus curves with this IOL, values at the main foci are slightly below those reported by Cano-Ortíz et al. (17), but this cannot be attributed to the existence of SSNG in one of the eyes.

**Figure 6:**
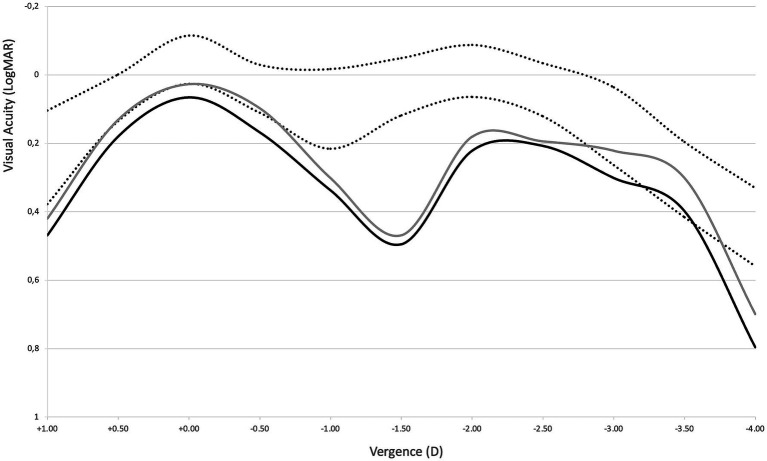
Defocus curves for the affected (solid black line) and fellow control eye (solid gray line). Area between dotted lines represents the ±SD range reported by Palomino-Bautista et al. (18) for monocular defocus curves with this IOL as a reference.

Monocular defocus curves show almost identical profiles for both the affected and control eyes (Figure 6), confirming the findings of the optical bench analysis on the explanted IOLs. They also show, however, that the profiles are reduced compared with the range reported by Palomino-Bautista et al. (18) for monocular defocus curves with this IOL, represented in the figure as the area between the dotted lines.

### 6.7 Optical coherence tomography

**Figure 7:**
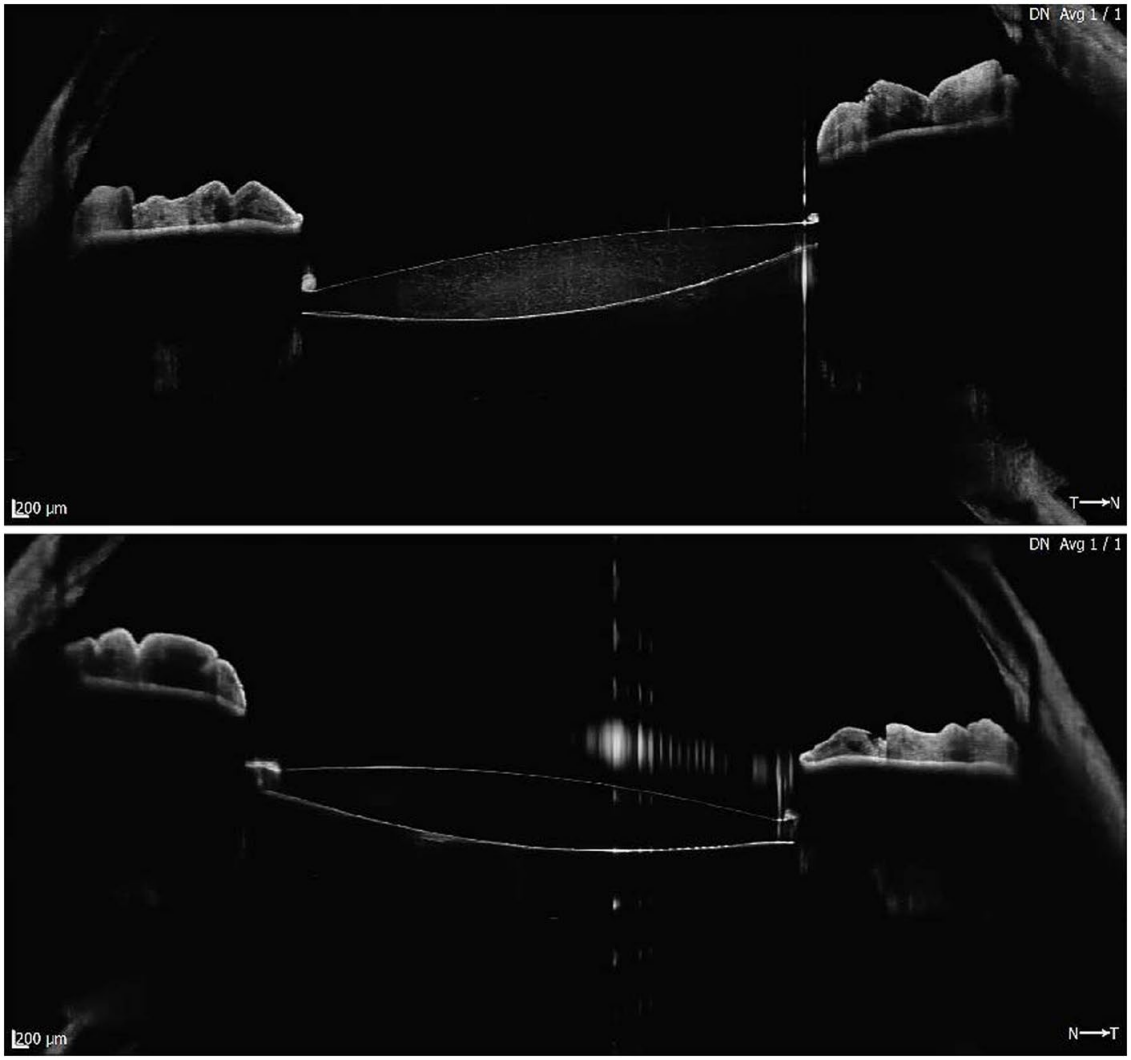
OCT image of the affected eye (above) and the fellow eye (below). Speckle noise within the body of the lens is evident in the above image and cannot be observed in the image below.

OCT images of the IOLs show visible differences between the affected eye and the fellow eye (Figure 7).

The affected eye image shows significantly more speckle noise within the lens body. In images of scattering tissue, speckle has a dual role as a source of noise and as a carrier of information about tissue microstructure (19); therefore, the images suggest that all the microstructure of the polymer might be affected in the IOLs presenting whitening, even though by the definition of loss of transparency it affects mainly the areas just below the surfaces.

### 6.8 Scheimpflug tomography

**Figure 8:**
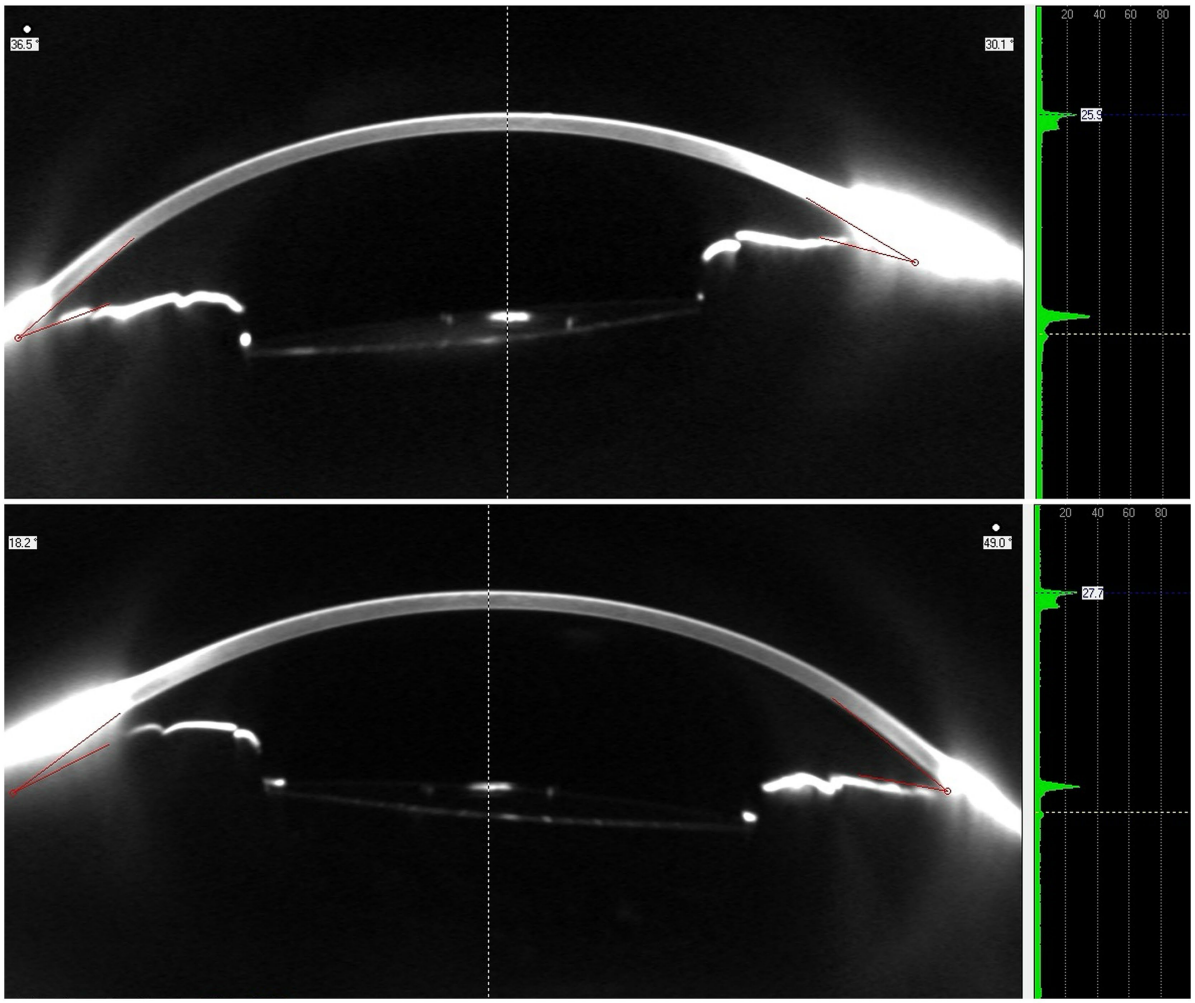
Scheimpflug tomographic image (left) and densitometry profile (right) of the affected eye (above) and the fellow eye (below).

Although the differences between both affected and control IOLs are not as evident as in OCT imaging, Scheimpflug tomographic images do show noticeable differences between the affected IOLs and control (Figure 8).

Densitometry analysis provided by the system shows a slightly higher peak for the anterior surface of the affected eye, as well as a more evident second peak corresponding to the posterior surface. These findings confirm that the higher optical density is mainly below the anterior surface of the lens.

### 6.9 Light distortion analysis

**Figure 9:**
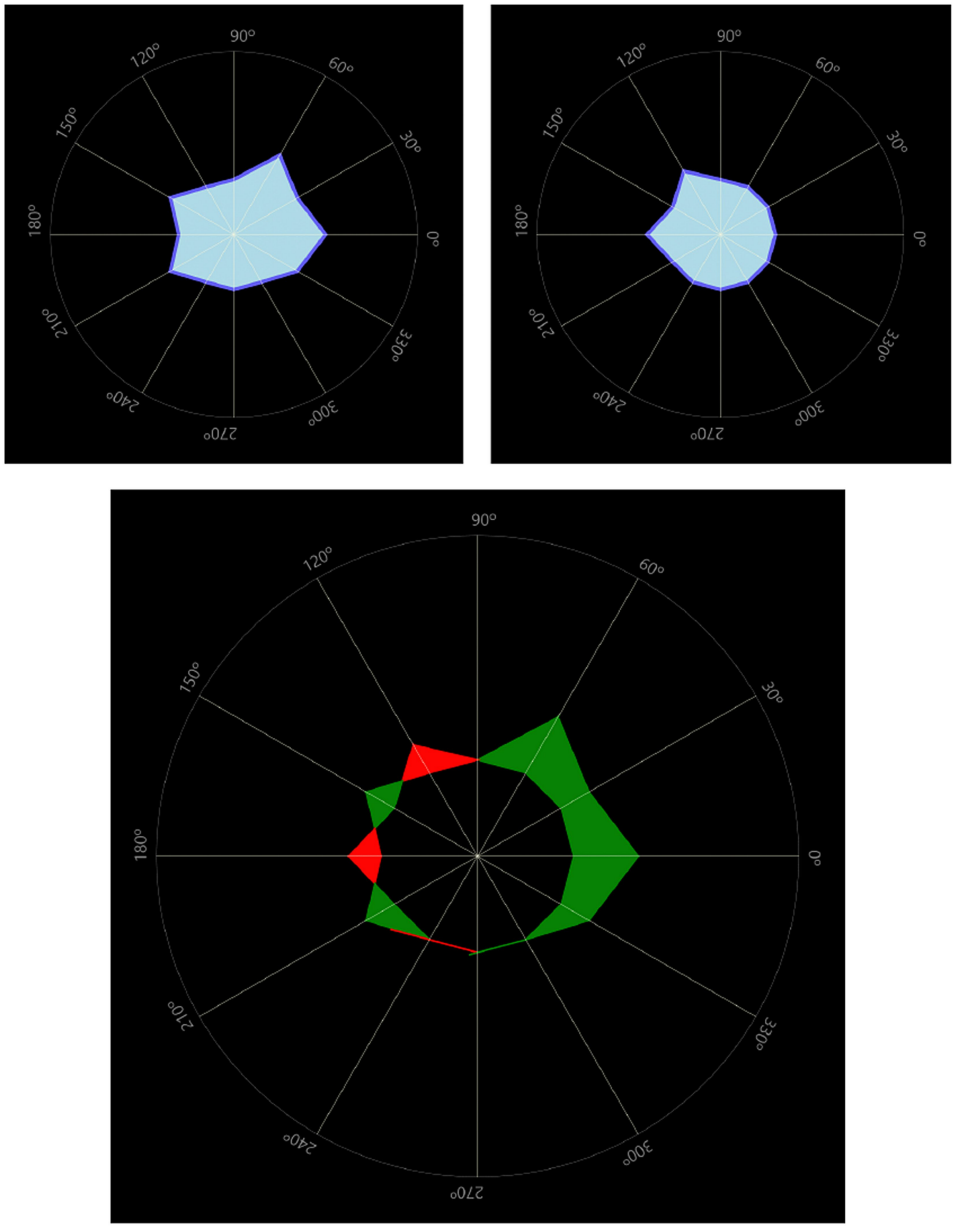
Light distortion comparison between the affected eye (top left) and the fellow eye (top right). The bottom image overlaps both distortion areas to compute the difference between eyes. In this case 30% larger in the affected eye. Light distortion indexes were 12.89% in the affected eye and 9.55% in the fellow eye.

Light distortion measures were found to be 30% greater in the affected eye than those in the fellow eye for one of the patients (Figure 9).

The other patient, however, showed almost identical light distortion values in both the affected and fellow eyes, being significantly higher than in the previous patient. Light distortion indexes in this case were 28.97% in the affected eye and 28.89% in the fellow eye.

### 6.10 Contrast sensitivity

Contrast sensitivity values were obtained in both patients that were affected by SSNG, showing a reduction in the area below the CSF curve of the affected eye compared to the fellow eye of 17 and 10%, respectively, with this loss being mainly for high frequencies. If considering reference ranges provided by the CSF systems for the general population, in one case both eyes were within normal range, falling just below normal range only for 18 cpd in the affected eye and in the other case with both affected and fellow eyes just below normal range.

**Figure 10:**
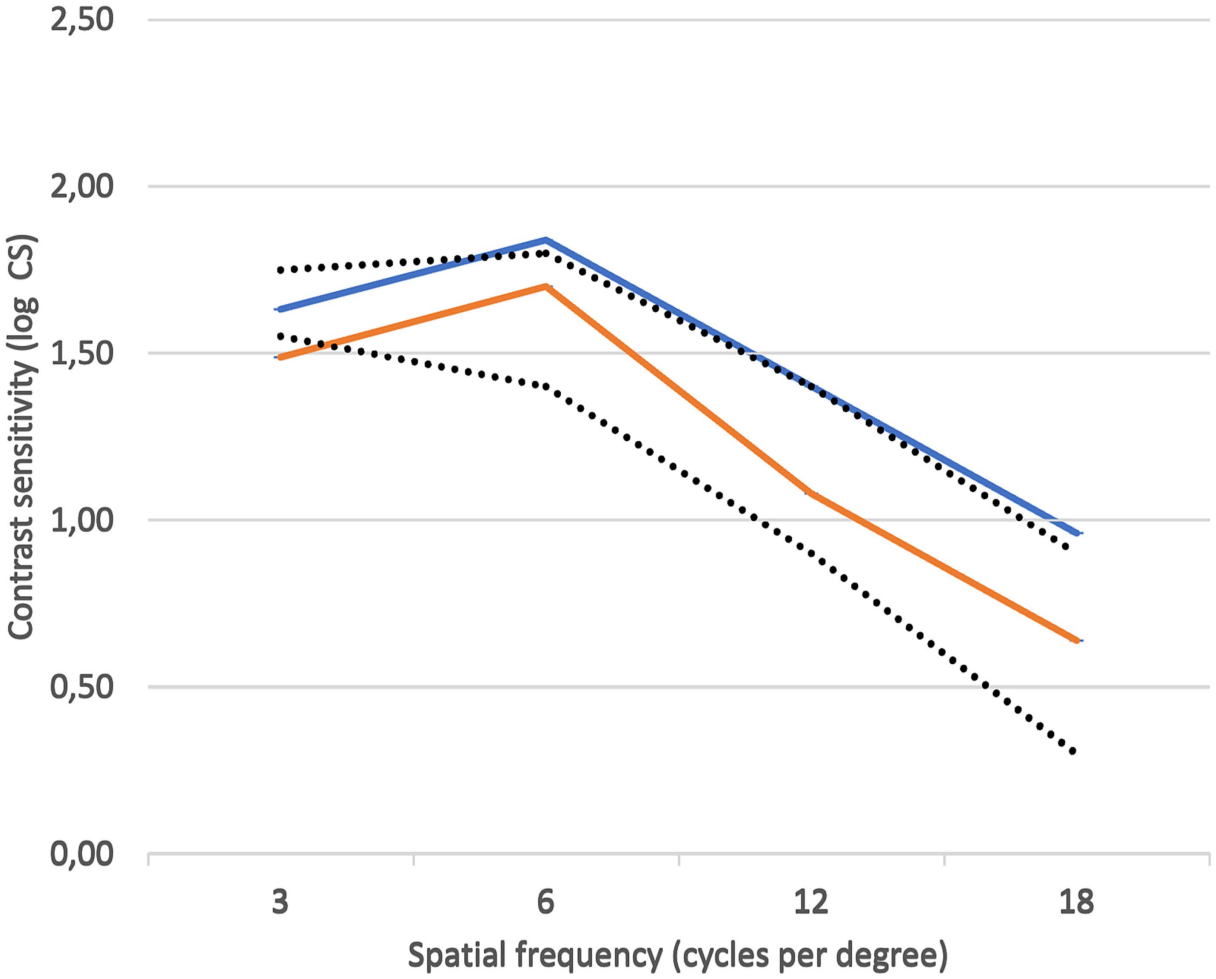
Contrast sensitivity plots of the affected eye (orange) and the fellow unaffected eye (blue). Reference normal range was that of 60 years of age or older, as determined by Escaf et al. (20).

Figure 10 shows the contrast sensitivity plots of both the affected and fellow unaffected eyes in the 73-year-old patient. Although values are slightly decreased in the affected eye compared to the fellow eye, they both fall within the normal range established for over 60 years of age by Escaf et al. (20).

### 6.11 Patient-reported outcomes

Both patients note haziness differences when alternating eye closure and discomfort in intense direct light. Questionnaire responses indicate no difficulty in most activities, with some issues with night vision and driving at dusk. Overall satisfaction with vision is high, although one patient reported some difficulties in daily life and bothersome hazy vision. Both report frequent, moderately severe halos and starbursts.

## 7 Discussion

It has been observed that certain types of hydrophobic acrylic intraocular lenses may undergo a transformation over time, leading to a whitish appearance due to an elevation in light scattering on their surface (6, 13). This study provides an *in vivo* and *in vitro* analysis of the only three cases reported to date of IOL whitening in a new hydrophobic IOL material. The study aimed to elucidate the potential causes of whitening and characterize its nature and impact, both *in vivo* and *in vitro*. Although the study failed to provide potential causes, it did provide valuable insights into the nature and impact of IOL whitening.

It has been previously reported that, in explanted hydrophobic IOLs with the presence of SSNG, the transmission of light within the visible range experiences little reduction compared with an unused control lens of the same material, does not show calcium phosphate deposits, and has no evidence of hydrolysis, and the loss of transparency disappears in dehydrated state but reappears after rehydration (1, 12, 13). The lab findings observed in the explanted IOL analysis here reported confirm these items; the whitening observed and the fact that it disappears in a dehydrated state, allow establishing SSNG as the most likely reason.

The microvacuoles producing the light scattering that characterizes the “whitening” of SSNG are water molecules infiltrating the subsurface of the IOLs in the polymer structure, forming aggregates with diameters below 0.5 μm (between 140 and 185 nm) (6, 21), enough size to scatter visible light (15).

The observations from the analysis of Scheimpflug images confirm the increased backscatter of the IOLs with SSNG compared to the control IOLs, with densitometry profiles showing higher optical density on both IOL surfaces, with microvacuoles distributed more toward the surface of the IOLs (22). Utilizing scanning electron microscopy, Ong et al. (23) identified SSNG microvacuoles up to 120 μm beneath the surface.

The analysis of the OCT images suggests, however, that changes in the polymer structure affect the whole body of the lens. The level of speckle noise detected in the image of the IOLs affected by SSNG resembles the appearance typically associated with a more hydrophilic acrylic material when observed through OCT (24). Speckle obtained from OCT has been shown to be *signal-carrying* and used to infer about the *microstructure* of the tissue (11). Jesus DA et al. used statistical modeling of OCT speckle to extract this information from OCT images and infer corneal properties (25).

There is a certain controversy in the literature with regard to the visual impact of glistening and SSNG. Beheregaray et al. reported reductions in VA and CSF correlated with the increase in intraocular light scatter generated by SSNG, but the values obtained were within the normal range for the given age (26).

The outcomes obtained in the cases analyzed in the present study do agree with these findings, showing a little reduction in visual performance in the affected eyes but not differing significantly from the fellow eye or the norm; hence, the reduction in visual performance in these cases cannot be completely attributed to the presence of SSNG.

More recently, hydrophobic IOLs were associated with a significantly greater amount of SSNG and glistening than other materials 15 to 20 years after implantation but without visual impact in patients (10). This is not always the case, since cases where significant subjective visual loss due to SSNG 5 years after implantation, alongside starbursts, glare and cloudy vision, were also reported in the literature, although explantation was not considered necessary in this case (27), or significant visual loss 10 years after implantation where the IOLs were indeed explanted (7).

Light distortion generated by SSNG was assessed in the present study using the LDA system. The values obtained in both the affected and fellow eyes are higher than the average values obtained in previous reports in patients implanted with this IOL, but fall within the range of values reported (28) and therefore cannot be attributed fully to SSNG. In any case, these values are still lower than those reported in the literature by different authors with other multifocal IOLs using the same method (29, 30).

The presence of SSNG in the IOLs analyzed here was detected shortly after implantation, which is a significant difference from previous reports. Although both patients reported haloes and starbursts with little visual impact or concern, these are more likely attributed to the diffractive pattern of the trifocal design rather than an effect of SSNG-induced scatter, agreeing with previous reports with the same IOL in uneventful patients (17, 18, 31, 32). Haziness can be directly attributed to SSNG, and one of the patients did report it as bothersome and of severe intensity, although there was no difficulty in carrying out any of the tasks included in the CATQuest9SF questionnaire.

To date, the cause of SSNG occurring after implantation is still unknown. The most likely cause seems to be of chemical nature, although the fact that the cases discussed in the present study only occurred in one of the eyes of each of the patients affected makes it difficult to establish a hypothesis.

It has been described that certain serum components can reduce tension on the interface between aqueous humor and the IOLs. Particularly, the presence of phospholipids in high concentrations in the aqueous humor, such as when the hematoaqueous barrier function is broken, acts as detergents that reduce surface tension at the IOL–aqueous humor interface (33), which could facilitate the formation of microvacuoles that lead to SSNG.

The findings of the analysis here reported allow confirming SSNG as the most likely cause for the loss of transparency in these cases. It must be noted that only one eye was affected in each patient, which facilitates patient awareness due to comparison between eyes, particularly so when the affected lens is a premium IOL and patient expectations are high. A follow-up is desirable to assess any changes with time.

Given that the clinical impact of SSNG in non-explanted cases was objectively mild, with only a slight reduction in visual performance and low impact on patient-reported outcomes, the decision to explant and exchange an IOL affected by SSNG should be individualized. This decision should be made in conjunction with the patient, taking into account their subjective visual disturbances, daily visual demands, and expectations. In any case, these are the first three cases of SSNG reported with this new IOL material out of more than 20,000 lenses implanted to date (according to the manufacturer). This low incidence confirms that, although an infrequent event, SSNG can occur in any hydrophobic IOL. The clinical impact of SSNG in non-explanted cases can be objectively considered as low, with a mild effect on visual performance and low impact on PROs.

## Data Availability

The datasets presented in this article are available by the authors upon reasonable request. Requests to access the datasets should be directed to alejandro.cervino@uv.es.
